# Spironolactone versus placebo, bisoprolol, and doxazosin to determine the optimal treatment for drug-resistant hypertension (PATHWAY-2): a randomised, double-blind, crossover trial

**DOI:** 10.1016/S0140-6736(15)00257-3

**Published:** 2015-11-21

**Authors:** Bryan Williams, Thomas M MacDonald, Steve Morant, David J Webb, Peter Sever, Gordon McInnes, Ian Ford, J Kennedy Cruickshank, Mark J Caulfield, Jackie Salsbury, Isla Mackenzie, Sandosh Padmanabhan, Morris J Brown, The British Hypertension Society's PATHWAY Studies Group

**Affiliations:** a Institute of Cardiovascular Sciences University College London and National Institute for Health Research (NIHR) UCL/UCL Hospitals Biomedical Research Centre, London, UK; b Medicines Monitoring Unit, Medical Research Institute, University of Dundee, Dundee, UK; c Clinical Pharmacology Unit, University of Edinburgh, Centre for Cardiovascular Science, Queen's Medical Research Institute, Edinburgh, UK; d International Centre for Circulatory Health, Imperial College, London, UK; e BHF Glasgow Cardiovascular Research Centre, Institute of Cardiovascular and Medical Sciences, University of Glasgow, Glasgow, UK; f Robertson Centre for Biostatistics, Glasgow University, Glasgow, UK; g Cardiovascular Medicine & Diabetes, King's College London, London, UK; h William Harvey Research Institute, Barts and The London School of Medicine and Dentistry, Queen Mary University of London, London, UK; i Clinical Pharmacology Unit, Addenbrooke's Hospital, University of Cambridge, UK

## Abstract

**Background:**

Optimal drug treatment for patients with resistant hypertension is undefined. We aimed to test the hypotheses that resistant hypertension is most often caused by excessive sodium retention, and that spironolactone would therefore be superior to non-diuretic add-on drugs at lowering blood pressure.

**Methods:**

In this double-blind, placebo-controlled, crossover trial, we enrolled patients aged 18–79 years with seated clinic systolic blood pressure 140 mm Hg or greater (or ≥135 mm Hg for patients with diabetes) and home systolic blood pressure (18 readings over 4 days) 130 mm Hg or greater, despite treatment for at least 3 months with maximally tolerated doses of three drugs, from 12 secondary and two primary care sites in the UK. Patients rotated, in a preassigned, randomised order, through 12 weeks of once daily treatment with each of spironolactone (25–50 mg), bisoprolol (5–10 mg), doxazosin modified release (4–8 mg), and placebo, in addition to their baseline blood pressure drugs. Random assignment was done via a central computer system. Investigators and patients were masked to the identity of drugs, and to their sequence allocation. The dose was doubled after 6 weeks of each cycle. The hierarchical primary endpoints were the difference in averaged home systolic blood pressure between spironolactone and placebo, followed (if significant) by the difference in home systolic blood pressure between spironolactone and the average of the other two active drugs, followed by the difference in home systolic blood pressure between spironolactone and each of the other two drugs. Analysis was by intention to treat. The trial is registered with EudraCT number 2008-007149-30, and ClinicalTrials.gov number, NCT02369081.

**Findings:**

Between May 15, 2009, and July 8, 2014, we screened 436 patients, of whom 335 were randomly assigned. After 21 were excluded, 285 patients received spironolactone, 282 doxazosin, 285 bisoprolol, and 274 placebo; 230 patients completed all treatment cycles. The average reduction in home systolic blood pressure by spironolactone was superior to placebo (–8·70 mm Hg [95% CI −9·72 to −7·69]; p<0·0001), superior to the mean of the other two active treatments (doxazosin and bisoprolol; −4·26 [–5·13 to −3·38]; p<0·0001), and superior when compared with the individual treatments; versus doxazosin (–4·03 [–5·04 to −3·02]; p<0·0001) and versus bisoprolol (–4·48 [–5·50 to −3·46]; p<0·0001). Spironolactone was the most effective blood pressure-lowering treatment, throughout the distribution of baseline plasma renin; but its margin of superiority and likelihood of being the best drug for the individual patient were many-fold greater in the lower than higher ends of the distribution. All treatments were well tolerated. In six of the 285 patients who received spironolactone, serum potassium exceeded 6·0 mmol/L on one occasion.

**Interpretation:**

Spironolactone was the most effective add-on drug for the treatment of resistant hypertension. The superiority of spironolactone supports a primary role of sodium retention in this condition.

**Funding:**

The British Heart Foundation and National Institute for Health Research.

## Introduction

Resistant hypertension, defined as suboptimal blood pressure control despite treatment with at least three blood pressure-lowering drugs, is associated with a poor prognosis. This is caused by organ damage from prolonged exposure to suboptimal blood pressure control, and to the association with diabetes, chronic kidney disease, and obesity.^1^, ^2^ The prevalence of resistant hypertension is estimated to be at least 10% of treated hypertensive patients, which would equate to a potential prevalence of about 100 million people globally.^1^, ^3^ There has been a growing perception that controlling blood pressure in resistant hypertension is beyond the reach of existing drug therapies, leading to the emergence of device-based therapies such as renal denervation and baroreceptor stimulation.

**Research in context**

**Evidence before this study**

International guidelines have converged on a definition of resistant hypertension as blood pressure that is not controlled to target despite treatment with three recommended blood pressure-lowering drugs at maximum tolerated doses—namely, an angiotensin-converting-enzyme (ACE) inhibitor or an angiotensin II receptor blocker (ARB; “A”), plus a calcium channel blocker (CCB; “C”), plus a thiazide-like diuretic (“D”)—ie, A + C + D. We searched MEDLINE, Embase, and the Cochrane CENTRAL register, for English language publications published up to July, 2015, for randomised controlled trials, and open and observational studies, of the drug treatment of resistant hypertension. This review was cross-referenced to the National Institute for Health and Care Excellence (NICE) hypertension clinical guideline review of drug treatment of resistant hypertension (CG 127) from 2011 and a recently published meta-analysis of randomised controlled trials and non-randomised studies of drug treatment of resistant hypertension with aldosterone antagonists. The meta-analysis suggested that spironolactone can be an effective blood pressure-lowering treatment for patients with resistant hypertension, however, the NICE review concluded that the quality of existing evidence was low. Three randomised controlled trials, of 135 patients (combined), had shown spironolactone to be superior to the placebo in reducing seated clinic blood pressure, when added to existing treatment for resistant hypertension. But there had been no previous randomised controlled trials comparing spironolactone with other blood pressure-lowering drugs to determine if it is the most effective treatment for resistant hypertension.

**Added value of this study**

PATHWAY-2 is, to our knowledge, the first randomised controlled trial to compare different blood pressure-lowering treatments in rigorously assessed patients with resistant hypertension, and the first comparison of mineralocorticoid receptor blockade with alternative recommended classes that block the sympathetic nervous system (α blockers and β blockers). The size, crossover design, and hierarchical primary endpoints of PATHWAY-2 enabled demonstration at high significance (p<0·0001) that spironolactone 25–50 mg/day is by far the most effective drug added to A + C + D, for the treatment of resistant hypertension; blood pressure was controlled (home systolic blood pressure <135 mm Hg) in 60% of patients. The role of sodium retention in causing resistant hypertension was strongly suggested by a low baseline plasma renin, despite treatment with three drugs which usually elevate renin, and by a significant inverse correlation between renin and blood pressure reduction by spironolactone. The individual crossover data show that spironolactone is the most effective add-on drug, by a large margin, in the overwhelming majority of patients confirmed as adherent, but resistant, to treatment with A + C + D.

**Implications of all the available evidence**

The unequivocal superiority of spironolactone, together with supportive efficacy and safety data from longer term observational studies, should influence treatment guidelines globally. Whether the superiority is specific for mineralocorticoid receptor blockade, reflecting overlap between primary aldosteronism and resistant hypertension, will require comparison of spironolactone with other types of diuretic. Meanwhile, truly resistant hypertension can now be considered rare and redefined as blood pressure not controlled by A + C + D + spironolactone.

For drug treatment of resistant hypertension, international guidelines specifically refer to fourth-line therapy for patients, whose blood pressure is not controlled by treatment with three drugs, typically A + C + D, where “A” is an angiotensin-converting-enzyme (ACE) inhibitor or an angiotensin II receptor blocker (ARB), “C” is a calcium channel blocker (CCB), and “D” is a thiazide or thiazide-like diuretic.^4^, ^5^, ^6^, ^7^ The choice of fourth-line drug treatment for resistant hypertension has been entirely empirical, reflecting an absence of data from prospective randomised controlled trials comparing different drug treatment options. The underlying pathophysiological basis for resistant hypertension is also poorly understood.

One hypothesis is that resistant hypertension is predominantly caused by sodium retention, due in part to the reduced doses of diuretics prescribed in recent years; if so, drugs with a diuretic action would be the most effective additional treatment.^8^, ^9^ An alternative hypothesis is that resistant hypertension is a heterogeneous state, with average responses in study cohorts masking substantial individual patient differences. In the latter case, treatment could be stratified by use of biomarkers of sodium/volume status, particularly plasma renin level, to which sodium status is inversely related.^10^

We selected spironolactone as the drug with diuretic action (through blocking the mineralocorticoid receptor) because of observational and limited randomised controlled trial data suggesting good blood pressure-lowering efficacy in resistant hypertension, recently summarised in a meta-analysis.^11^ However, spironolactone has not been compared with alternative drugs recommended for resistant hypertension. It has therefore been unknown whether spironolactone is the most effective treatment, and if so, whether this applies to a subset of patients or the majority. We aimed to compare spironolactone with alternative fourth-line treatments targeting different pathogenetic mechanisms: the α1-adrenoceptor blocker, doxazosin, acting to reduce peripheral resistance, and the β1-adrenoceptor blocker, bisoprolol, which inhibits the release of renin, and reduces cardiac output. Our primary aim was to determine, for the first time, whether spironolactone is overall the most effective add-on drug treatment for resistant hypertension. The second aim was to determine whether plasma renin levels predict the most effective treatment for individual patients, and whether spironolactone would be most effective in patients with a low plasma renin as a marker of sodium retention. We therefore designed a randomised crossover trial so that each patient's best drug and its predictors could be discovered.

## Methods

### Study design and participants

In this 12-month double-blind, placebo-controlled, crossover phase 4 trial, patients were enrolled from 12 secondary care and 2 primary care sites in the UK. The protocol has been published.^12^ The trial enrolled patients aged 18–79 years with seated clinic systolic blood pressure 140 mm Hg or greater (or ≥135 mm Hg for patients with diabetes) and home systolic blood pressure (18 readings over 4 days) 130 mm Hg or greater, despite treatment for at least 3 months with maximally tolerated doses of three drugs. These had to be an ACE inhibitor or an ARB; “A”), a CCB (“C”), and diuretic (“D”). A full list of eligibility and exclusion criteria is provided in the appendix (pp 3,4). Special emphasis was given to assessment of adherence to the patient's baseline medication before randomisation by measurement of home systolic blood pressure 6 h after directly observed therapy, by returned tablet counts, and by measurement of serum ACE activity.

All patients gave informed written consent. The protocol was approved by Cambridge South Ethics Committee. There was no data monitoring board.

### Randomisation and masking

The full trial protocol is summarised in the appendix (p 15). After a month's single-blind placebo run-in, patients rotated through four cycles of once daily oral treatment with: (1) spironolactone 25–50 mg, (2) doxazosin modified release 4–8 mg, (3) bisoprolol 5–10 mg, and (4) placebo. The complete set of permutations of the sequence order for the four treatments in the crossover design were randomly ordered within blocks using computer generated pseudo random numbers. Study sites received the allocation for a particular participant by accessing a web-based randomisation system within the Robertson Centre for Biostatistics, Glasgow, Scotland, UK. The study drugs were masked by re-encapsulation at the Royal Free Hospital Pharmacy. Investigators and patients were masked to the identity of drugs, and to their sequence allocation.

### Procedures

The treatment cycles were initiated for 6 weeks at the lower dose, followed by forced titration to twice this dose for a further 6 weeks (total of 12 weeks). Patients unable to tolerate a drug in a cycle were allowed to move to the next drug in sequence. There was no washout period between the four treatment cycles (three active, one placebo). The entire study, including placebo run-in, lasted 1 year. After this, patients were invited to participate in a further 12-week cycle of open-label amiloride 10 mg, titrated to 20 mg after 6 weeks.

After initial screening and enrolment, there were nine subsequent visits on blinded medication: one after the placebo run-in, the remaining eight after the 6-weekly periods on each of the two doses of the three active drugs and placebo.

The primary endpoint measurement was average home systolic blood pressure, recorded in the morning and the evening in triplicate, on 4 consecutive days before study visits. For analysis, a maximum of the last 18 recordings for each measurement period—ie, days 2–4—if all completed, were used. A minimum of six blood pressure recordings per measurement period was required for a valid measurement of the home systolic blood pressure average. The home systolic blood pressure average for the primary endpoint included all of the aforementioned measurements throughout each treatment cycle—ie, at week 6 and week 12). For seated clinic blood pressure, the mean of the last two measurements was recorded as the clinic blood pressure. The home and clinic blood pressures were measured for every patient using the approved, automated blood pressure monitor (WatchBP Home, Microlife, Clearwater, FL, USA), which was allocated to the patient for their sole use for the duration of the trial. Patients were instructed by the specialist nurses in self-measurement of blood pressure and technique was visibly re-enforced at each visit, when the research nurse measured patients' blood pressure using the same monitor.

Plasma renin was measured at baseline (following run-in on background “A + C + D” and placebo) with a Diasorin Liaison automated chemiluminescent immunoassay for direct renin mass.^13^ Serum electrolytes were measured at every visit.

### Outcomes

The primary objective was to test the hypothesis that spironolactone is the most effective add-on treatment for patients with resistant hypertension. The primary analysis used an average of home systolic blood pressure recorded throughout the treatment cycle. We prespecified hierarchical primary endpoints; (1) the difference in the home systolic blood pressure between spironolactone and placebo, followed if significant by (2) the difference in home systolic blood pressure between spironolactone and the average of the other two active drugs, (doxazosin and bisoprolol), followed if significant by (3) the difference in home systolic blood pressure between spironolactone and each of the other two active drugs.

The secondary objectives included evaluation of; (1) clinic blood pressure responses to randomised treatments; (2) blood pressure control rates—ie, home systolic blood pressure less than 135 mm Hg; (3) whether plasma renin concentrations and other baseline characteristics could help personalise treatment by predicting the best drug treatment; and (4) adverse event rates during each treatment cycle.

To test the hypothesis that plasma renin (measured on a background of three drugs—ie, A + C + D), will predict the most effective fourth-line drug, we examined the relationship between plasma renin and the reduction of home systolic blood pressure with each drug, adjusted for the placebo response. We also identified the best treatment for each patient—ie, the one on which they achieved the lowest blood pressure, and estimated for each drug the relationship between baseline renin and the likelihood that it would provide the best response. Adverse events were recorded at each visit.

### Statistical analysis

The sample size was estimated to be 294 patients, based on detecting a difference of 3 mm Hg (SD 12) in home systolic blood pressure between each of the experimental drugs and the placebo treatment, with 90% power using a single sample *t* test at the 0·003 significance level (this was chosen in order that the 0·01 level could be adjusted for three planned comparisons). However, the hierarchical analysis subsequently adopted negated the need to adjust p values.

We tested hypotheses with the mixed effect models to analyse continuous variables, with unstructured covariances for repeated measures within a patient. We included prespecified baseline covariates (sex, age, height, weight, smoking history, and the baseline value of the outcome being analysed) in the models. Least squares means for each treatment estimated from these models are presented. Blood pressure control and response rates were analysed with logistic regression models, which also included the baseline covariates. Comparisons of adverse event rates between treatments were done with χ^2^ tests and Fisher's exact p values are given. The probability that a drug would provide the best response as a function of baseline renin, was estimated from multinomial logistic regression.

Intention-to-treat analyses excluded only those participants with no primary outcome data at any follow-up visit (21 patients). Other participants with missing data were included, and we assumed that data was missing at random (ie, its absence was unrelated to the unobserved value).

Analyses were done with SAS (Cary, USA) version 9.3.

The trial is registered with EudraCT number 2008-007149-30, and ClinicalTrials.gov number, NCT02369081.

### Role of the funding source

The funders had no role in the data collection, data analysis, or data interpretation, or the writing of the report. The investigators and all authors had sole discretion in the data analysis and interpretation, writing of the report, and the decision to submit for publication. The corresponding author had full access to all of the data and the final responsibility to submit for publication.

## Results

**Figure 1:**
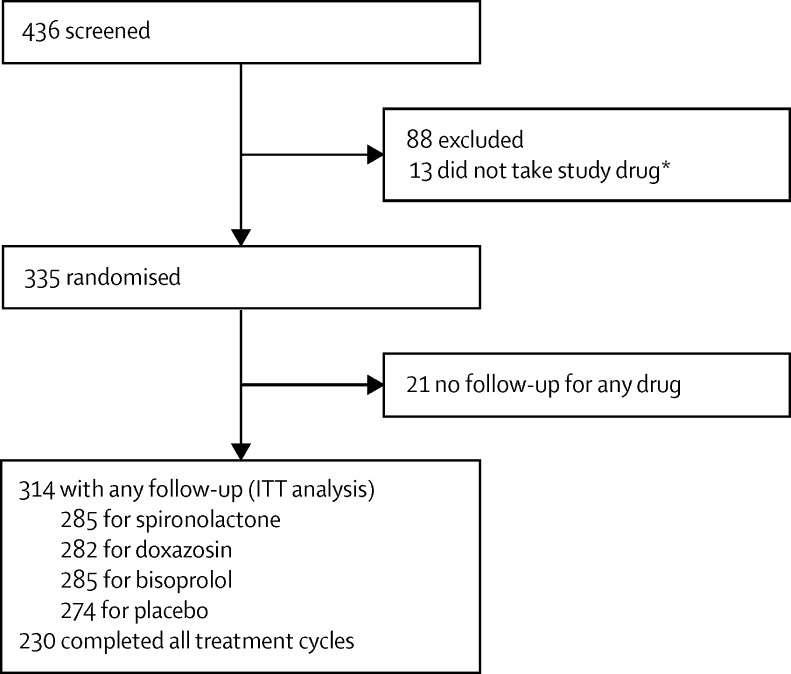
Trial profile *Randomised but instructed not to take any study drug after the result of directly observed therapy. Participants with any follow-up were included in the intent-to-treat analysis and the full analysis dataset consisted of all available data for these participants. Per-protocol analyses included participants who completed all follow-up visits without major deviation from the protocol. ITT=intention to treat.

Between May 15, 2009, and July 8, 2014, we screened 436 patients and randomised 335, of which 21 had no follow-up for any drug and were excluded from the intention-to-treat analysis, which comprised 314 patients who had any follow-up data. 285 patients received spironolactone, 282 doxazosin, 285 bisoprolol, and 274 placebo; 230 patients completed all treatment cycles (figure 1; appendix p 15). Last patient visit was on June 5, 2015. Table 1 shows the baseline of characteristics of the randomised patients.

**Figure 2:**
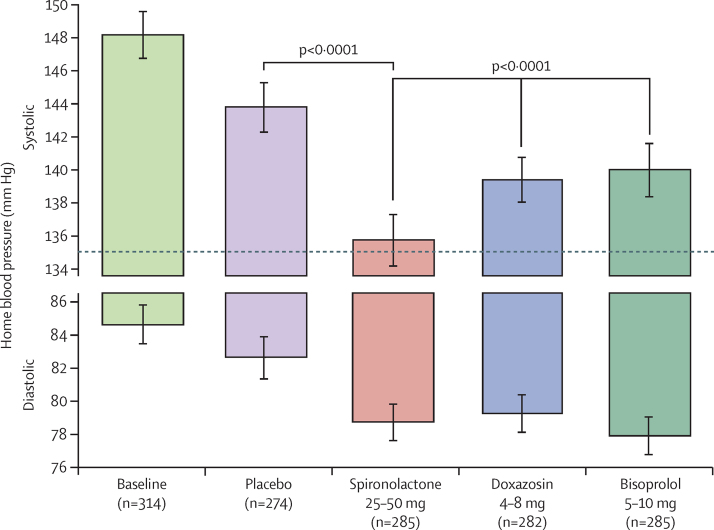
Home systolic and diastolic blood pressures comparing spironolactone with each of the other cycles The top and bottom of each column represents the unadjusted home systolic and diastolic blood pressures, respectively, averaged across the mid-cycle (low-dose) and end-of-cycle (high-dose) visits (6 weeks and 12 weeks) in which patients received the drug. Error bars represent 95% CI. Comparisons are as described under methods for the primary endpoint.

The average reduction in home systolic blood pressure throughout the treatment cycle with spironolactone was superior to each of: placebo (–8·70 mm Hg [95% CI −9·72 to −7·69]; p<0·0001); the mean of the other two active treatments (doxazosin and bisoprolol, −4·26 [–5·13 to 3·38]; p<0·0001); and each of the other individual treatments; doxazosin (–4·03 [–5·04 to 3·02]; p<0·0001) and bisoprolol (–4·48 [–5·50 to −3·46]; p<0·0001; figure 2; Table 2, Table 3, Table 4).

The differences in favour of spironolactone were greater when restricted to home systolic blood pressure values measured on the higher doses at the end of each treatment cycle (table 3). Spironolactone showed the largest difference between high and low doses (table 4); this was true irrespective of which treatment was assigned in the previous cycle. In further, prespecified sensitivity analyses, similar differences in favour of spironolactone were seen in 230 patients who received all four study drugs and 216 patients on three “A + C + D” background medications (appendix pp 5–8). The steep dose response for spironolactone, and superiority over other treatments, were seen also in a parallel group analysis of the first treatment cycle (appendix p 17).

The results for seated clinic systolic blood pressure largely mirror those seen with home systolic blood pressure except that there was a large placebo effect on clinic blood pressure that was not seen with home blood pressure measurement (appendix p 9). Full details of all blood pressure data (home and clinic), including diastolic pressures and heart rate are shown in appendix (p 10).

Overall 219 (68·9% [95% CI 63·6–73·8]) of 314 patients achieved target home systolic blood pressure of less than 135 mm Hg. The comparison of control rates is shown in the appendix (p 11). 58% of patients had their blood pressure controlled with spironolactone, which was superior to rates for other treatments. Most patients who were controlled by doxazosin or bisoprolol had a still greater fall in blood pressure on spironolactone, which was consequently the most effective treatment in almost 60% of patients. This was at least three times the proportion in whom doxazosin or bisoprolol were the most effective.

**Figure 3:**
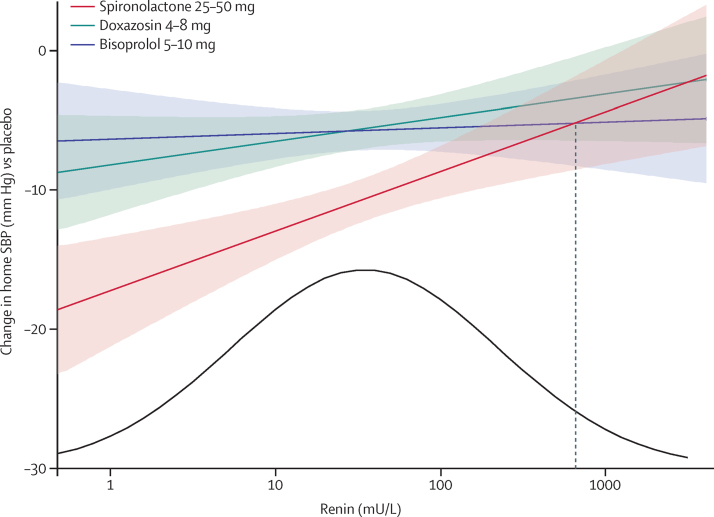
Blood pressure response versus renin Regression (90% CI) of placebo corrected change in home systolic blood pressure versus renin for spironolactone (*r*^2^=0·037, p=0·003), doxazosin (*r*^2^=0·007, p=0·183), and bisoprolol (*r*^2^=0·0004, p=0·750). Blood pressures were averaged across the mid-cycle and end-of-cycle visits (6 and 12 weeks) for every treatment cycle. The distribution curve is fitted to the baseline renins observed in the study. The vertical dashed line shows that the blood pressure fall on bisoprolol numerically exceeds that on spironolactone only in the top 3% of the renin distribution. A more detailed histogram for plasma renin is shown in the appendix (p 20).

The proportion of patients in whom spironolactone was their best drug for blood pressure lowering was most evident on the planned analyses of prediction by plasma renin of blood pressure response to each drug and the likelihood that different drugs would be best at different points in the plasma renin distribution. Figure 3 shows the relation between plasma renin (measured at baseline whilst patients were receiving their usual medication—ie, A + C + D) and the blood pressure-lowering response to each active treatment corrected for the placebo effect. There was a clear inverse relation between the home systolic blood pressure fall with spironolactone and plasma renin, not seen with bisoprolol or doxazosin. Moreover, the blood pressure response to spironolactone was superior to bisoprolol and doxazosin across most of the plasma renin distribution (figure 3). Only in a small minority of patients, with very high plasma renin levels, did the mean home systolic blood pressure response to doxazosin or bisoprolol overlap that to spironolactone. Analysis of each patient's best drug clearly showed that, although spironolactone was the best blood pressure-lowering treatment throughout almost the entire renin distribution, the likelihood of being superior, and the magnitude of this superiority, was several-fold higher for spironolactone than the other drugs at the lower end of the distribution (figure 3; appendix pp 18,19).

**Figure 4:**
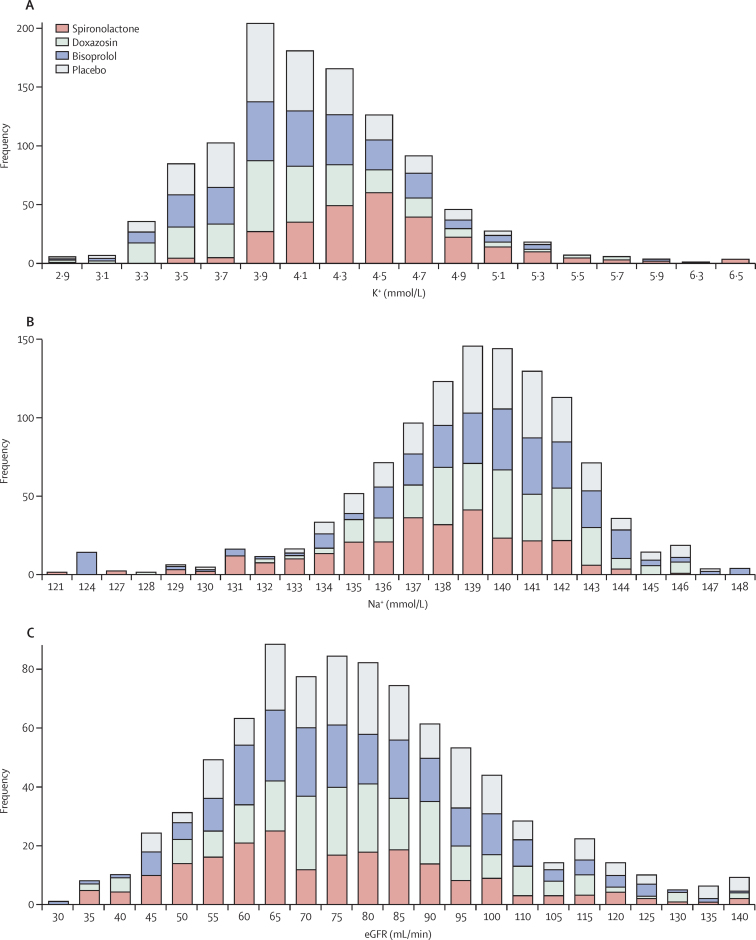
Distribution of potassium (A), sodium (B), and estimated glomerular filtration rate (eGFR; C) on each drug Values on the x axis are the measurement at the end of each 12-week cycle, and the y axis represents the number of patients with values in each bin on the x axis.

All active treatments were well tolerated with similar low rates of adverse events and withdrawals due to adverse events (table 5; appendix p 12 shows the numbers and rates of the commonest adverse events, and appendix p 13 shows all serious adverse events). Notably, discontinuations due to renal impairment, hyperkalaemia, and gynaecomastia were not increased with spironolactone relative to other treatments and placebo. Serum sodium was reduced with spironolactone (–1·91 mmol/L, −1·35%), but not the other treatments, whereas some increase in potassium levels was observed with both bisoprolol and spironolactone (appendix p 14). Serum creatinine levels increased and estimated glomerular filtration rate (eGFR) decreased (appendix p 14). Stacking of the distribution of serum sodium, potassium, and eGFR at the end of the treatment cycle for each drug shows that all drugs lowering blood pressure in this population cause some fall in eGFR, and that the frequency of abnormal numbers for each parameter is low (figure 4). None of these was clinically serious or led to withdrawals from the trial. Only six (2%) of 285 patients exposed to spironolactone developed a serum potassium on a single occasion greater than 6·0 mmol/L, with a maximum of 6·5 mmol/L.

## Discussion

PATHWAY-2 is the first randomised controlled trial to compare spironolactone with other blood pressure-lowering drug treatments in a well-characterised population of patients with resistant hypertension. The study shows that spironolactone was by far the most effective blood pressure-lowering treatment for patients with resistant hypertension. This was true in terms of the magnitude of the blood pressure response, the proportion of patients achieving a stringent measure of blood pressure control (home systolic blood pressure <135 mm Hg), and the proportion in whom it was more effective than either of the non-diuretic alternative drugs. These findings suggest that the predominant underlying pathophysiological cause of resistant hypertension is sodium retention, despite existing baseline diuretic therapy. This conclusion is supported by our finding that the response to spironolactone had a clear inverse relation with plasma renin, was especially effective at lower plasma renin levels, and yet the most effective drug throughout the range of plasma renin.

Bisoprolol and doxazosin were more effective than placebo at reducing blood pressure as so-called add-on therapy for resistant hypertension, but significantly less effective than spironolactone. Thus, this study has for the first time, established a clear hierarchy for drug treatment of resistant hypertension in which spironolactone is the most effective add-on therapy (ie, fourth-line drug in addition to A + C + D) for most patients. Bisoprolol or doxazosin are less effective alternatives for those intolerant of spironolactone.

PATHWAY-2 is the first study to use home blood pressure averages rather than clinic blood pressure to assess the primary outcome of blood pressure response in patients with resistant hypertension. This is important because home blood pressure measurement reduces the placebo effect, as was evident in our study, and eliminated patients whose blood pressure could have been spuriously elevated at baseline due to so-called white coat hypertension. Indeed, we noted a large placebo effect on clinic systolic blood pressure readings, exceeding 10 mm Hg (table 3), which points to important confounding in interpreting data from previous studies that have relied on clinic blood pressure readings alone to assess the efficacy of drug and non-drug-based interventions in these patients. The magnitude of home systolic blood pressure reduction with spironolactone versus placebo (–8·7 mm Hg) in the present study is consistent with data from two smaller randomised controlled trials versus placebo that have used ambulatory blood pressure monitoring (secondary analysis) to measure changes in mean daytime pressures (–7·31 mm Hg).^11^, ^14^, ^15^ The reduction in seated clinic systolic blood pressure with spironolactone (–20·7 mm Hg) was also similar to the reduction in clinic blood pressure from meta-analysis of previous small randomised contolled trials versus placebo (–24·3 mm Hg) and single arm studies with no comparator (–22·7 mm Hg).^11^

Spironolactone substantially increased the likelihood of achieving blood pressure control relative to bisoprolol or doxasosin, with almost 60% achieving blood pressure control within 3 months of starting treatment. This challenges the concept that resistant hypertension cannot be treated adequately with existing drug therapies, a concept that might have contributed to the growth of non-drug-based therapies such as renal denervation. Indeed, only 15 of 285 patients assessed on spironolactone failed to achieve a home systolic blood pressure lower than 150 mm Hg (equivalent to a clinic systolic blood pressure of roughly 160 mm Hg), the usual eligibility criterion for denervation. Furthermore, it is clear from our data that spironolactone, unlike bisoprolol or doxazosin, exhibited a significant dose response with regard to the magnitude of blood pressure lowering. A previous crossover comparison of spironolactone with even higher doses (ie, 50–100 mg) in patients without resistant hypertension, also showed a dose response,^16^ suggesting that the highest dose of spironolactone used in our study (ie, 50 mg), might not be at the top of the dose range, and hence the potential for even better control rates with higher doses.

The superior response to spironolactone, compared with the other drugs, particularly in patients at the lower end of the distribution of plasma renin, supports the hypothesis that the predominant cause of resistant hypertension is sodium retention. The fact that spironolactone was the most effective drug across a wide range of baseline plasma renin values does not negate the hypothesis, because one would expect plasma renin levels to be elevated in patients receiving treatment with A + C + D, all of which usually increase plasma renin levels. Indeed, because plasma renin is substantially affected by background antihypertensive treatment, the interpretation and recognition of so-called low renin status has been uncertain in such populations. The median renin in PATHWAY-2, 34 mU/L, is roughly three times higher than that in the PATHWAY-1 study, of 600 patients with untreated hypertension (unpublished). However, 34 mU/L is a lower median than expected if the sole influence on baseline renin was drug treatment.^10^ It is probable that plasma renin in resistant hypertension is relatively suppressed by sodium retention, even though absolute values appear normal or high.

One contributor to the sodium retention could be under-dosing of background diuretic treatment. An alternative or additional possibility is that some patients with resistant hypertension have undetected aldosterone producing adenomas (APAs). Recent studies have shown that specific somatic mutations in the adrenal gland can result in micro-APAs, which are difficult to detect by conventional imaging.^17^ Additionally, some high-renin patients will have an element of secondary aldosteronism, explaining why spironolactone retains some efficacy at the upper end of the renin distribution. Determination of whether spironolactone is particularly effective treatment for resistant hypertension because it antagonises the effects of aldosterone will require a head-to-head comparison of spironolactone with an increase in dose of the background diuretic.

As well as being the most effective treatment for resistant hypertension, spironolactone was well tolerated. The doses of spironolactone used in the present study are low compared with the 200–400 mg daily used in other clinical circumstances. The 25–50 mg daily doses in PATHWAY-2 are consistent with previous studies of resistant hypertension in which 25 mg was the most common daily dose.^11^ These studies have shown that the main biochemical effects associated with spironolactone treatment are a reduction in serum sodium and an increase in potassium. We noted a magnitude of change (–1·19 mmol/L in sodium and 0·45 mmol/L in potassium) very similar to that reported in summary data from previous studies.^11^ Despite almost 14% of our patients having type 2 diabetes, only six patients receiving spironolactone developed potassium levels in excess of 6·0 mmol/L, which was detected by our routine monitoring and had no clinical consequence. We also recorded reductions in eGFR with all active blood pressure-lowering treatments that most likely reflect a reduction in renal perfusion pressure with blood pressure lowering. Thus, although spironolactone is both very effective and safe in resistant hypertension, it is important to monitor electrolytes (especially potassium) and renal function during the weeks after initiation of treatment, after dose escalation and periodically thereafter. A recent large longitudinal population study of the use of spironolactone showed no evidence of any increased incidence of admission to hospital or outpatient hyperkalaemia.^18^ Another recognised adverse effect of spironolactone treatment relates to its anti-androgen effects and the development of gynaecomastia, which has been reported to occur in roughly 6% of men.^11^, ^19^ We did not observe any cases in the present study, but this most likely reflects the relatively short duration of our study (3 months exposure).

The PATHWAY-2 study has some limitations. 3 months treatment exposure is relatively short. Nevertheless, observational studies of longer spironolactone treatment duration for resistant hypertension suggest that the magnitude of the initial blood pressure response is durable and that among adverse events, only gynaecomastia is exposure dependent.^11^, ^19^ The results of open-label treatment with amiloride (10–20 mg daily) during the run-out phase of our study will be reported later and will help to determine whether amiloride is an effective alternative to spironolactone. Our study excluded patients with an eGFR less than 45 mL/min, as have previous studies, thus there are no data on the safety profile of spironolactone in patients with resistant hypertension and an eGFR less than 45 mL/min. Our study included predominantly white Caucasian patients, thus it is unclear whether the results are applicable to other ethnic groups, however, spironolactone has been shown to be just as effective in a small observational study that included black American patients.^20^ The absence of washout periods, inherent in a study design already 1 year in length, might be considered a concern. However, we were confident from our previous crossover studies^16^ that carry-over would not be a problem, and are supported by the sensitivity analyses, including the absence of change in blood pressure during the placebo cycle, and similarity of the primary outcome result to the retrospective parallel group analysis of cycle 1 alone. At worst, we could have under estimated the superiority of spironolactone. Finally, the study does not include data for morbidity and mortality outcomes but blood pressure lowering is a powerful surrogate for clinical benefit, especially in this high-risk group of patients.

Our study also has a number of strengths. The patients in this study were particularly well characterised as resistant hypertension, with the use of home blood pressure monitoring to exclude so-called white coat hypertension, standardisation of background medication (A + C + D), directly observed therapy to exclude patients non-compliant with background medication, measurement of serum ACE to allow retrospective confirmation of the expected difference in distribution between patients receiving ACE inhibitor or ARB as one of their background drugs, and oversight by national specialist hypertension centres to exclude secondary hypertension. In the late stages of the study, we incorporated a new assay for monitoring all commonly administered antihypertensive drugs in patients' urine,^21^ and will report results from this substudy that strongly support a high adherence rate among our patients. Another strength was the design of the study, which incorporated a random cross over design, allowing each patient's best drug to be determined, and predictive testing of this to be assessed. Mechanistic haemodynamic substudies, which could add to the predictive value of renin and help us to understand the pathophysiology of resistant hypertension, were included at most sites and will be reported separately.^12^ Finally, this was the first randomised controlled trial directly comparing different active drug treatments in resistant hypertension and it produced an unequivocal result.

The results of PATHWAY-2 have broad international relevance because of convergent guideline recommendations, which recommend A + C + D as the preferred three-drug combination at step 3.^22^ Our finding, that spironolactone was clearly the most effective treatment for resistant hypertension, should influence future treatment guidelines and clinical practice globally. The finding could indeed stimulate an early redefinition of resistant hypertension to include a trial of spironolactone before the label is applied. A longer-term question is whether the antecedent to resistant hypertension is under treatment or wrong treatment, with the resistance to conventional drugs marking a subpopulation in whom spironolactone should be used at an earlier stage.

## Figures and Tables

**Table 1:** Baseline characteristics of the patients randomised into the PATHWAY-2 study (n=335)

		**Mean (SD) or N (%)**
Age (years)		61·4 (9·6)
Sex		
	Male	230 (69%)
	Female	105 (31%)
Weight (kg)		93·5 (18·1)
Smoker		26 (7·8%)
Home		
	Systolic blood pressure (mm Hg)	147·6 (13·2)
	Diastolic blood pressure (mm Hg)	84·2 (10·9)
	Heart rate (beats per min)	73·3 (9·9)
Clinic		
	Systolic blood pressure (mm Hg)	157·0 (14·3)
	Diastolic blood pressure (mm Hg)	90·0 (1·5)
	Heart rate (beats per min)	77·2 (12·2)
24 h urine (mmol/24 h)		
	Sodium	137·1 (71·8)
	Potassium	70·5 (29·5)
Blood electrolytes (mmol/L)		
	Sodium	139·6 (3·0)
	Potassium	4·1 (0·5)
eGFR (mL/min)		91·1 (26·8)
Diabetic		46 (14%)

eGFR=estimated glomerular filtration rate.

**Table 2:** Home systolic blood pressure averaged across both visits for each cycle

	**Blood pressure (mm Hg)**	**Change from baseline (mm Hg)**
**Mean**		
Spironolactone	134·9 (134·0 to 135·9)	−12·8 (−13·8 to −11·8)
Doxazosin	139·0 (138·0 to 140·0)	−8·7 (−9·7 to −7·7)
Bisoprolol	139·4 (138·4 to 140·4)	−8·3 (−9·3 to −7·3)
Placebo	143·6 (142·6 to 144·6)	−4·1 (−5·1 to −3·1)
**Mean differences**		
Spironolactone *vs* placebo	8·70 (−9·72 to −7·69)	p<0·0001
Spironolactone *vs* mean bisoprolol and doxazosin	−4·26 (−5·13 to −3·38)	p<0·0001
Spironolactone *vs* doxazosin	−4·03 (−5·04 to −3·02)	p<0·0001
Spironolactone *vs* bisoprolol	−4·48 (−5·50 to −3·46)	p<0·0001

Data are mean (95% CI). Home systolic blood pressure throughout the treatment cycle for each drug (includes data from mid-cycle at week 6 and the final visit at week 12). Least squares means from mixed effects models adjusted for baseline covariates. Hierarchical primary endpoints each tested only if the preceding tests were significant.

**Table 3:** Home systolic blood pressure at final visit of each cycle

	**Blood pressure (mm Hg)**	**Change from baseline (mm Hg)**
**Mean**		
Spironolactone	133·5 (132·3 to 134·8)	−14·4 (−15·6 to −13·1)
Doxazosin	138·8 (137·6 to 140·1)	−9·1 (−10·3 to −7·8)
Bisoprolol	139·5 (138·2 to 140·8)	−8·4 (−9·7 to −7·1)
Placebo	143·7 (142·5 to 145·0)	−4·2 (−5·4 to −2·9)
**Mean differences**		
Spironolactone *vs* placebo	−10·2 (−11·7 to −8·74)	p<0·0001
Spironolactone *vs* mean bisoprolol and doxazosin	−5·64 (−6·91 to −4·36)	p<0·0001
Spironactone *vs* doxazosin	−5·30 (−6·77 to −3·83)	p<0·0001
Spironolactone *vs* bisoprolol	−5·98 (−7·45 to −4·51)	p<0·0001

Data are mean (95% CI). Sensitivity analysis using only the mean home systolic blood pressure at the final visit of each cycle (week 12).

**Table 4:** Home systolic blood pressure dose response (higher *vs* lower dose)

	**Blood pressure (mm Hg)**	**p value**
Spironolactone	−3·86 (−5·28 to −2·45)	<0·0001
Doxazosin	−0·88 (−2·32 to 0·56)	0·23
Bisoprolol	−1·49 (−2·94 to −0·04)	0·04
Placebo	−0·68 (−2·10 to 0·75)	0·35

Difference in mean home systolic blood pressure after treatment with the lower (week 6) and higher doses (week 12) of each treatment.

**Table 5:** Adverse events and withdrawals

	**Spironolactone**	**Doxazosin**	**Bisoprolol**	**Placebo**	p value*
Serious adverse events	7 (2%)	5 (2%)	8 (3%)	5 (2%)	0·82
Any adverse event	58 (19%)	67 (23%)	68 (23%)	42 (15%)	0·036
Withdrawals for adverse events	4 (1%)	9 (3%)	4 (1%)	3 (1%)	0·28

Data are n (%).

* p values for Fisher's exact test. The most common adverse events in at least 5% of patients on any treatment are shown in appendix p 12.
