# Supplementary material for: Spironolactone versus placebo, bisoprolol, and doxazosin to determine the optimal treatment for drug-resistant hypertension (PATHWAY-2): a randomised, double-blind, crossover trial
Source: Lancet. 2015 Nov 21;386(10008):2059–68. doi: 10.1016/S0140-6736(15)00257-3 (PMC4655321; doi:10.1016/S0140-6736(15)00257-3)
Supplement: Supplementary appendix [file mmc1.pdf]

THE LANCET

Supplementary appendix

This appendix formed part of the original submission and has been peer reviewed. We post it as supplied by the authors.

Supplement to: Williams B, MacDonald TM, Morant S, et al. Spironolactone versus placebo, bisoprolol, and doxazosin to determine the optimal treatment for drug-resistant hypertension (PATHWAY-2): a randomised, double-blind, crossover trial. *Lancet* 2015; published online Sept 21. http://dx.doi.org/10.1016/S0140-6736(15)00257-3.

Online Appendix

Contents	Title	Page
Pathway Studies Group		2
Supplementary Table S1	Eligibility criteria	3
Supplementary Table S2	Exclusion criteria	4
Supplementary Table S3	Home SBP responses in 230 patients who received both doses of each drug	5
Supplementary Table S4	Home SBP responses in 216 patients who received 3 background drug	7
Supplementary Table S5	Clinic Systolic BP	9
Supplementary Table S6	Home and Clinic BP (systolic and diastolic) & Heart Rate	10
Supplementary Table S7	BP Control Rates	11
Supplementary Table S8	Adverse Events	12
Supplementary Table S9	Serious adverse events	13
Supplementary Table S10	Changes in serum sodium, potassium and creatinine and eGFR	14
Supplementary Figure S1	Study Schema	15
Supplementary Figure S2	Detailed Study Flow Diagram	16
Supplementary Figure S3	Home Systolic Blood Pressures at Baseline and End of Cycle 1	17
Supplementary Figure S4	Prediction by plasma renin of best drug	18
Supplementary Figure S5	Frequency histogram for baseline plasma renin	19

***PATHWAY Studies Group**

1. Anne Schumann, Jo Helmy, Carmela Maniero, Timothy J Burton, Ursula Quinn, Lorraine Hobbs, Jo Palmer (Addenbrooke's Cambridge, UK)
2. David Collier, Nirmala Markandu, Manish Saxena, Anne Zak, Enamuna Enobakhare (William Harvey Institute, QMUL, London, UK)
3. Judith Mackay, Simon A McG Thom, Candida Coughlan, (Imperial College Health Care NHS Trust, London, UK)
4. Alison R McGinnis, Evelyn Findlay (Ninewells Hospital & Medical School, Dundee, UK)
5. Adrian G Stanley, Gerry P McCann, Christobelle White, Peter Lacy, Caroline J. Gardiner-Hill, Sheraz Nazir, Pankaj Gupta. (Glenfield Hospital, Leicester, UK)
6. Sandosh Padmanabhan, Gordon T McInnes, Scott Muir, Linsay McCallum (Glasgow Cardiovascular Research centre, Glasgow, UK)
7. Vanessa Melville, Iain M MacIntyre (University of Edinburgh Western General Hospital, Edinburgh, UK)
8. Handrean Soran, See Kwok, Karthirani Balakrishnan. (Old Saint Mary's Hospital, Manchester, UK)
9. Richard D'Souza (Exeter Hospital, Exeter, UK)
10. Richard Hobbs, Rachel Iles. (Primary Care Clinical Sciences University Birmingham, Birmingham, UK)
11. Khin Swe Myint (Clinical Research and trials Unit, University of East Anglia, Norwich, UK)
12. John Cannon, Sue Hood (Ixworth GP Practice, Ixworth, UK)
13. Krzysztof Rutkowski (Cardiovascular Medicine & Diabetes, King's College London, London, UK)
14. Una Martin (University Hospital Birmingham, Birmingham, UK)
15. Sharon Kean, Robbie Wilson, Richard Papworth (Robertson Centre for Biostatistics and Glasgow CTU, Glasgow, UK)

Supplementary Table S1

Eligibility criteria

1. Patients aged 18-79 years†
2. Patients will all have hypertension that is not controlled to target: clinic systolic BP ≥ 5 mmHg above target (i.e. ≥ 140 mmHg for non-diabetic hypertensives or ≥ 135 mmHg for diabetics), under one of the following conditions:
a) Treatment for at least 3 months with lisinopril 20 mg (A) + amlodipine 10 mg (C)+ bendroflumethiazide 2.5 mg (D) or their equivalents‡
b) Patients who have received the three drugs or equivalents specified in a), and are either intolerant to one category, or tolerate only a lower dose (e.g. amlodipine 5 mg or lisinopril 10 mg)
c) Patients receiving the three drugs or equivalents specified in a), who are receiving additional drugs for their hypertension, may be included if the investigator
1) feels it is appropriate to stop these additional drugs at the screening visit and
2) anticipates that the BP criteria for inclusion will be met when re-checked at the baseline visit
3. Patients with a home systolic BP average of >130 mmHg or within 15mmHg of clinic BP over the 4 days prior to the baseline visit.

† Patients aged 79 years at the time of screening visit, due to turn 80 years old prior to randomisation are eligible for the study
‡ The three months of prior treatment may include substitution of an existing A (=ACEi, ARB, or direct renin inhibitor), C (=CCB) or D (=Diuretic - any except spironolactone) for an 'equivalent' dose of another drug in the same category. Such a substitution may be made at the screening visit if the investigator considers this appropriate. If the dose of one of the background drugs would be suboptimal in combination with spironolactone, because of the potential for electrolyte imbalance, the PI may elect to reduce that dose at screening. The reduced dose must then be continued unchanged throughout the study. This scenario is most likely in patients with plasma potassium at the upper end of the normal range.

Supplementary Table S2. Exclusion criteria

1.	Inability to give informed consent;	
2.	Participation in a clinical study involving an investigational drug or device within 4 weeks of screening;	
3.	Secondary or accelerated hypertension;	
4.	Type 1 diabetes;	
5.	eGFR<45 mls/min;	
6.	Plasma potassium outside of normal range on two successive measurements during screening;	
7.	Pregnancy, planning to conceive, or women of child-bearing potential, i.e. not using barrier effective contraception;	
8.	Anticipated change of medical status during the trial (e.g. surgical intervention requiring >2 weeks convalescence);	
9.	Absolute contra-indication to study drugs (e.g. asthma) or previous intolerance of trial therapy;	
10.	Sustained atrial fibrillation;	
11.	Recent (<6 months) cardiovascular event requiring hospitalisation (e.g. myocardial infarction or stroke);	
12.	Suspected non-adherence to antihypertensive treatment (see above);	
13.	Requirement for study drug for reason other than to treat hypertension, (e.g. β-blockers for angina or diuretics other than those to treat hypertension);	
14.	Current therapy for cancer;	
15.	Concurrent chronic illness, or other reasons likely to preclude 40 week participation in the study;	
16.	Clinic Systolic BP >200 mmHg or diastolic BP >120mmHg, with PI discretion to override if home BP measurements are lower	
17.	Any concomitant condition that, in the opinion of the investigator, may adversely affect the safety and/or efficacy of the study drug or severely limit that patients life-span or ability to complete the study (e.g. alcohol or drug abuse, disabling or terminal illness, mental disorders);	
18.	Treatment with any of the following medications;	
	a.	Oral corticosteroids within 3 months of screening. Treatment with systemic corticosteroids is also prohibited during study participation;
	b.	Chronic stable use, or unstable use of NSAIDs (other than low dose aspirin) is prohibited. Chronic use is defined as >3 consecutive or non-consecutive days of treatment per week. In addition intermittent use of NSAIDs is strongly discouraged throughout the study and NSAIDs if required, must not be used for more than a total of 2 days. For those requiring analgesics during the study, paracetamol is recommended.
	c.	The use of short acting nitrates (e.g. sublingual nitroglycerin) is permitted. However, participants should not take short acting oral nitrates within 4 hours of screening or an subsequent visit;
	d.	The use of long acting nitrates (e.g. Isordil) is permitted but the dose must be stable for at least 2 weeks prior to screening and randomisation;
	e.	The use of sympathomimetic decongestants is permitted , however, not within 1 day prior to any study visit/BP assessment;
	f.	The use of theophylline is permitted but the dose must be stable for at least 4 weeks prior to screening and throughout the study;
	g.	The use of phosphodiesterase type V inhibitors is permitted; however study participants must refrain from taking these medications for at least 1 day prior to screening or any subsequent study visits;
	h.	The use of alpha-blockers is not permitted, with the exception of afluzosin and tamsulosin for prostatic symptoms
19.	A pill count will be made at the end of the 4 week run-in period and those with adherence <70% will be excluded from randomisation	

Supplementary Table S3. Home SBP responses in 230 patients who received both doses of each drug

		Blood pressure (mmHg)	Change from baseline
Means	Spironolactone	135.2 (134.2,136.3)	-12.7 (-13.7,-11.6)
	Doxazosin	139.6 (138.5,140.7)	-8.3 (-9.4, -7.3)
	Bisoprolol	139.8 (138.7,140.9)	-8.1 (-9.2, -7.0)
	Placebo	144.4 (143.3,145.5)	-3.5 (-4.6, -2.4)
			p value
Mean differences	Spironolactone vs Placebo (1)	-9.15 (-10.2,-8.14)	<.001
	Spironolactone vs mean Bisoprolol/Doxazosin (2)	-4.46 (-5.34,-3.57)	<.001
	Spironolactone vs Doxazosin (3)	-4.34 (-5.36,-3.32)	<.001
	Spironolactone vs Bisoprolol (3)	-4.57 (-5.60,-3.54)	<.001

A. Average HSBP for both visits on each drug

Home systolic BP throughout the treatment cycle for each drug (includes data from mid-cycle at week 6 and the final visit at week 12). Least squares means from mixed effects models adjusted for baseline covariates.

(1),(2),(3) Hierarchical primary endpoints each tested only if the preceding tests were significant.

Final visit on each drug		Blood pressure (mmHg)	Change from baseline
Means	Spironolactone	133.2 (131.9,134.5)	-14.9 (-16.2,-13.5)
	Doxazosin	139.5 (138.2,140.8)	-8.6 (-9.9, -7.3)
	Bisoprolol	139.7 (138.4,141.0)	-8.4 (-9.7, -7.1)
	Placebo	144.6 (143.3,145.9)	-3.4 (-4.8, -2.1)
			p value
Mean differences	Spironolactone vs Placebo	-11.4 (-12.9,-9.95)	<0·001
	Spironolactone vs mean Bisoprolol/Doxazosin	-6.38 (-7.66,-5.10)	<0·001
	Spironolactone vs Doxazosin	-6.28 (-7.75,-4.81)	<0·001
	Spironolactone vs Bisoprolol	-6.48 (-7.96,-5.01)	<0·001

B. Home Systolic Blood Pressure only at final visit on each drug

Least squares means from mixed effects models adjusted for baseline covariates.

Higher vs lower dose	Blood pressure (mmHg)	p value
Spironolactone	-4.54 (-5.98,-3.11)	<.001
Doxazosin	-0.90 (-2.35, 0.56)	0.227
Bisoprolol	-1.98 (-3.45,-0.50)	0.009
Placebo	0.13 (-1.30, 1.56)	0.856

C. Dose response: difference in mean home systolic BP after treatment with the lower (week 6) and higher doses (week 12) of each treatment.

Supplementary Table S4. Home SBP responses in 216 patients on three background drugs (eligibility criterion 2a in Table S1)*

Both visits on each drug		Blood pressure (mmHg)	Change from baseline
**Means**	Spironolactone	134.8 (133.6,136.0)	-12.6 (-13.8,-11.4)
	Doxazosin	138.4 (137.3,139.6)	-9.0 (-10.2, -7.8)
	Bisoprolol	138.5 (137.3,139.7)	-8.9 (-10.1, -7.7)
	Placebo	142.9 (141.8,144.1)	-4.5 (-5.6, -3.3)
			**p value**
**Mean differences**	Spironolactone vs Placebo [1]	-8.15 (-9.33,-6.96)	<.001
	Spironolactone vs mean Bisoprolol/Doxazosin [2]	-3.67 (-4.71,-2.64)	<.001
	Spironolactone vs Doxazosin [3]	-3.64 (-4.83,-2.45)	<.001
	Spironolactone vs Bisoprolol [3]	-3.71 (-4.91,-2.51)	<.001

A. Average HSBP for both visits on each drug

Home systolic BP throughout the treatment cycle for each drug (includes data from mid-cycle at week 6 and the final visit at week 12). Least squares means from mixed effects models adjusted for baseline covariates.

[1],[2],[3] Hierarchical primary endpoints each tested only if the preceding tests were significant.

* The average dose of the most commonly used background drugs were losartan 92.7 mg (usual maximal dose 100mg), amlodipine 8.3 mg (usual maximal dose 10mg), and bendroflumethiazide 3.1 mg (usual maximal dose 2.5mg).

Final visit on each drug		Blood pressure (mmHg)	Change from baseline
**Means**	Spironolactone	133.4 (131.9,134.9)	-14.3 (-15.8,-12.8)
	Doxazosin	138.2 (136.7,139.6)	-9.5 (-11.0, -8.0)
	Bisoprolol	138.7 (137.2,140.2)	-9.0 (-10.4, -7.5)
	Placebo	143.2 (141.7,144.7)	-4.4 (-5.9, -2.9)
			**p value**
**Mean differences**	Spironolactone vs Placebo	-9.85 (-11.6,-8.12)	<0·001
	Spironolactone vs mean Bisoprolol/Doxazosin	-5.04 (-6.54,-3.55)	<0·001
	Spironolactone vs Doxazosin	-4.78 (-6.50,-3.06)	<0·001
	Spironolactone vs Bisoprolol	-5.31 (-7.03,-3.59)	<0·001

B. Home Systolic Blood Pressure only at final visit on each drug

Least squares means from mixed effects models adjusted for baseline covariates.Sensitivity analysis using only the mean home systolic BP at the final visit of each cycle (week 12).

Higher vs lower dose	Blood pressure (mmHg)	p value
Spironolactone	-3.36 (-5.04,-1.69)	<.001
Doxazosin	-1.23 (-2.91, 0.45)	0.151
Bisoprolol	-1.33 (-3.02, 0.36)	0.123
Placebo	-0.04 (-1.69, 1.62)	0.965

C. Dose response: difference in mean home systolic BP after treatment with the lower (week 6) and higher doses (week 12) of each treatment.

Supplementary Table S5. Clinic SBP

		Blood pressure (mmHg)	Change from baseline
Means	Spironolactone	136·5 (134·4,138·7)	-20·7 (-22·9,-18·6)
	Doxazosin	141·0 (138·8,143·1)	-16·3 (-18·5,-14·2)
	Bisoprolol	141·0 (138·8,143·2)	-16·3 (-18·4,-14·1)
	Placebo	146·5 (144·3,148·6)	-10·8 (-13·0, -8·7)
			**P value**
Mean differences	Spironolactone vs Placebo	-9·92 (-11·3,-8·59)	<0·001
	Spironolactone vs mean Bisoprolol/Doxazosin	-4·44 (-5·59,-3·28)	<0·001
	Spironolactone vs Doxazosin	-4·42 (-5·75,-3·09)	<0·001
	Spironolactone vs Bisoprolol	-4·45 (-5·80,-3·11)	<0·001
High vs low dose	Spironolactone	-3·01 (-4·87,-1·15)	0·002
	Doxazosin	-0·55 (-2·44, 1·34)	0·568
	Bisoprolol	-0·47 (-2·38, 1·44)	0·631
	Placebo	-0·97 (-2·84, 0·90)	0·311

Seated clinic systolic BP throughout the treatment cycles (includes data from week 6 and the end of the cycle at week 12) for each drug. Least squares means from mixed effects models adjusted for baseline covariates

Supplementary Table S6. Home systolic BP and seated clinic systolic BP and heart rate – average for both visits on each drug

	Home		Clinic	
Systolic BP	Absolute	Change	Absolute	Change
Spironolactone	134·9 (134·0,135·9)	-12·8 (-13·8,-11·8)	136·5 (134·4,138·7)	-20·7 (-22·9,-18·6)
Doxasosin	139·0 (138·0,140·0)	-8·7 (-9·7, -7·7)	141·0 (138·8,143·1)	-16·3 (-18·5,-14·2)
Bisoprolol	139·4 (138·4,140·4)	-8·3 (-9·3, -7·3)	141·0 (138·8,143·2)	-16·3 (-18·4,-14·1)
Placebo	143·6 (142·6,144·6)	-4·1 (-5·1, -3·1)	146·5 (144·3,148·6)	-10·8 (-13·0, -8·7)
Diastolic BP				
Spironolactone	78·9 (78·3, 79·4)	-5·7 (-6·3, -5·1)	80·1 (78·8, 81·4)	-10·3 (-11·6, -9·0)
Doxasosin	79·5 (78·9, 80·0)	-5·1 (-5·7, -4·5)	81·0 (79·7, 82·3)	-9·4 (-10·7, -8·1)
Bisoprolol	78·3 (77·7, 78·9)	-6·3 (-6·9, -5·7)	78·9 (77·6, 80·2)	-11·5 (-12·8,-10·2)
Placebo	83·1 (82·6, 83·7)	-1·4 (-2·0, -0·9)	84·4 (83·1, 85·6)	-6·1 (-7·3, -4·8)
Heart rate (per min)				
Spironolactone	74·7 (74·0, 75·4)	0·7 (0·1, 1·4)	78·5 (77·1, 79·9)	0·8 (-0·6, 2·1)
Doxasosin	73·9 (73·2, 74·6)	-0·0 (-0·7, 0·6)	77·3 (75·9, 78·7)	-0·5 (-1·9, 0·9)
Bisoprolol	60·0 (59·3, 60·7)	-13·9 (-14·6,-13·2)	61·6 (60·2, 63·0	-16·1 (-17·5,-14·7)
Placebo	73·1 (72·4, 73·8)	-0·9 (-1·5, -0·2)	77·0 (75·6, 78·4)	-0·8 (-2·1, 0·6)

Absolute BPs are the means of values recorded at mid (6 weeks) and end of each treatment cycle (12 weeks). The change in BP and heart rate from baseline is also shown. Least squares means from mixed effects models adjusted for baseline covariates

Supplementary Table S7. Home systolic BP control rates

	HSBP		Patients	Met target		Least squares estimate (95% CI)	Odds ratio	p-value
	Baseline	Final	(n)	(r)	r/n (%)			
Control								
Spironolactone	148·3	133·9	282	163	57·8	58·0 (52·0, 63·7)		
Doxazosin	147·8	138·8	276	115	41·7	41·5 (35·8, 47·5)	0·52 (0·37, 0·73)	<·001
Bisoprolol	147·7	139·6	280	122	43·6	43·3 (37·5, 49·2)	0·55 (0·39, 0·78)	<·001
Placebo	147·8	143·5	270	66	24·4	23·9 (19·1, 29·4)	0·23 (0·16, 0·33)	<·001

BP control rates refer to patients achieving a home systolic BP of <135mmHg. Odds ratios from logistic regression models adjusted for baseline.

Supplementary Table S8. Adverse events

	Doxazosin		Bisoprolol		Spironolactone		Placebo		p value
	n	%	n	%	n	%	n	%	
Dizziness	36	6.0	72	12.2	36	6.1	26	4.5	0.091
Fatigue	10	3.3	18	6.1	22	7.4	9	3.1	0.283
Muscle spasms	20	6.6	5	1.7	3	1.0	0	0.0	<.001
Bradycardia	5	1.7	3	1.0	19	6.4	2	0.7	<.001
Dizziness postural	28	4.6	10	1.7	2	0.3	2	0.3	<.001
Nasopharyngitis	4	1.3	14	4.7	10	3.4	3	1.0	0.015
Oedema peripheral	0	0.0	13	4.4	4	1.3	2	0.7	<0.001
Diarrhoea	2	0.7	8	2.7	11	3.7	3	1.0	0.025
Dyspnoea exertional	1	0.3	9	3.1	4	1.3	0	0.0	0.002
Syncope	1	0.3	4	1.4	0	0.0	0	0.0	0.036
Tachycardia	0	0.0	6	1.0	0	0.0	0	0.0	0.029
Skin lesion	0	0.0	0	0.0	3	1.0	0	0.0	0.045

Distinct patients reporting adverse events with each preferred term. Terms listed are those that occurred in at least 5% of patients on any treatment, or were significantly different between treatments ($p<0.05$, Fisher's exact test)

Supplementary Table S8. Serious adverse events

Preferred term	Spironolactone	Doxazosin	Bisoprolol	Placebo
Any	7	5	8	5
Abdominal distension	0	0	1	0
Abdominal pain upper	0	0	0	1
Ankle fracture	0	0	1	0
Atrial fibrillation	1	0	0	0
Blood potassium increased	0	0	0	0
Bradycardia	0	0	1	0
Calculus ureteric	0	0	1	0
Cardiac pacemaker insertion	0	0	1	0
Chest pain	0	0	1	0
Cough	0	0	1	0
Diabetic neuropathy	0	0	1	0
Diarrhoea	1	0	0	0
Dizziness	0	0	1	0
Dyspnoea exertional	0	0	1	0
Fatigue	0	0	1	0
Gait disturbance	0	0	1	0
Hyperkalaemia	0	1	0	0
Hypertension	1	0	0	0
Intervertebral disc protrusion	0	0	0	1
Ischaemic stroke	0	0	0	0
Malaise	0	0	1	0
Myocardial infarction	0	0	0	0
Nausea	0	0	1	0
Neoplasm recurrence	1	0	0	0
Palpitations	0	1	0	0
Pelvic floor repair	0	0	0	0
Pulmonary oedema	0	1	0	0
Retinal haemorrhage	0	0	0	1
Skin cancer	0	0	0	1
Skin graft	1	0	0	0
Skin ulcer	1	0	0	0
Supraventricular tachycardia	0	1	0	0
Syncope	0	1	0	0
Transient ischaemic attack	0	0	1	0
Transitional cell carcinoma	1	0	0	0
Type 2 diabetes mellitus	1	0	0	1
Umbilical hernia repair	0	1	0	0
Urinary retention	0	0	1	0
Vision blurred	1	0	0	0
Vomiting	1	1	0	0

Distinct patients reporting serious adverse events with each preferred term

Supplementary Table 9. Changes in serum sodium, potassium and creatinine and eGFR

	Baseline	Follow up	Change	p value	Adjusted change*	p value
Sodium (mmol/L)						
Spironolactone	139·74	137·84	-1·91	<0·001	-1·89	<0·001
Doxazosin	139·37	139·30	-0·07	0·840	-0·17	0·634
Bisoprolol	139·96	139·78	-0·17	0·608	-0·06	0·873
Placebo	139·14	139·25	0·11	0·745	-0·08	0·816
Potassium (mmol/L)						
Spironolactone	4·06	4·49	0·43	<0·001	0·42	<0·001
Doxazosin	4·08	4·16	0·08	0·127	0·09	0·109
Bisoprolol	4·11	4·26	0·15	0·007	0·13	0·020
Placebo	4·05	4·09	0·03	0·410	0·06	0·281
Creatinine (µmol/L)						
Spironolactone	84·79	93·21	8·43	<0·001	7·18	<0·001
Doxazosin	83·75	91·04	7·28	0·003	7·46	<0·001
Bisoprolol	84·13	89·70	5·57	0·002	5·41	0·005
Placebo	79·73	81·85	2·12	0·094	4·35	0·036
eGFR (mls/min)						
Spironolactone	93·20	83·18	-10·02	0·004	-9·68	<0·001
Doxazosin	92·70	85·38	-7·32	0·023	-7·31	0·011
Bisoprolol	92·40	86·35	-6·05	0·006	-6·05	0·031
Placebo	92·52	92·67	0·15	0·923	-0·86	0·773

Baseline values and change at end of each treatment cycle for serum electrolytes, creatinine and estimated glomerular filtration rate (eGFR)
* Adjusted for change in Mean arterial blood pressure

Supplementary Figure 1. **Study Schema**

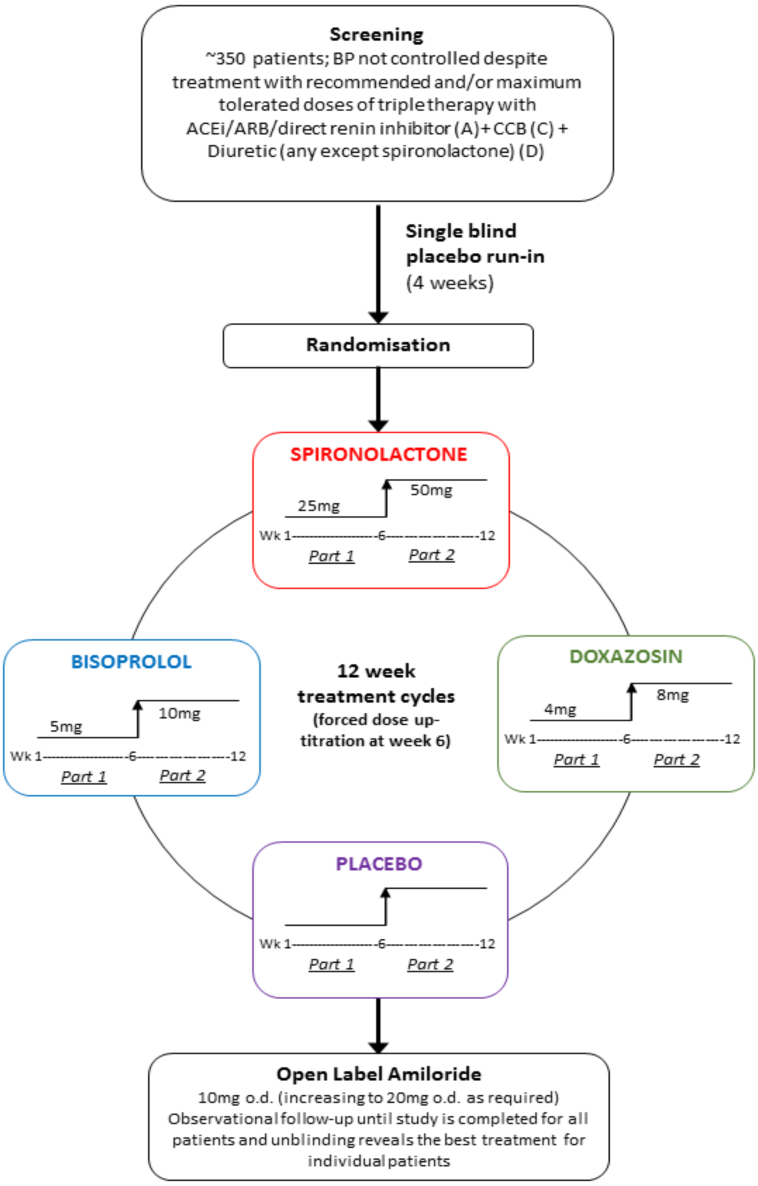

Supplementary Figure S2. Detailed Study Flow Diagram

436	assessed for eligibility
88	excluded
13	randomised but never exposed to study drug*
335	Intent to treat cohort

	Total	Spironolactone	Doxazosin	Bisoprolol	Placebo
**Phase 1**					
Intent to treat	335	86	83	85	81
Received study drug	333	86	83	85	79
With follow up	312	80	79	79	74
**Phase 2**					
Intent to treat	335	78	87	88	82
Received study drug	307	75	77	78	77
With follow up	303	73	75	78	77
**Phase 3**					
Intent to treat	335	82	87	83	83
Received study drug	282	71	72	76	63
With follow up	278	68	71	76	63
**Phase 4**					
Intent to treat	335	89	78	79	89
Received study drug	242	65	60	54	63
With follow up	233	64	57	52	60
**Overall**					
Intent to treat		335	335	335	335
Received study drug		297	292	293	282
Full analysis (all phases with follow up)		285	282	285	274
Per protocol (patients with follow up in all phases)		230	230	230	230

Reasons for non-completion	Spironolactone	Doxazosin	Bisoprolol	Placebo
Subject unwilling to continue	3	5	6	1
Lost to follow up	1	0	1	0
Adverse Event	4	8	4	3
Serious Adverse Event	0	1	0	0
Non-compliance	1	0	0	0
Subject violated protocol	0	1	0	1
Investigator terminated participation	6	1	2	1
Subject withdrawn consent but not use of data already collected	0	0	0	0
Subject withdrawn consent and use of data	0	0	0	0
Other Reason	3	2	4	4
Death	0	0	0	0

*Randomised but instructed not to take study medication because their SBP was <130 mmHg 6 hours after direct observation therapy.

Supplementary Figure S3. **Home Systolic Blood Pressures at Baseline and End of Cycle 1**

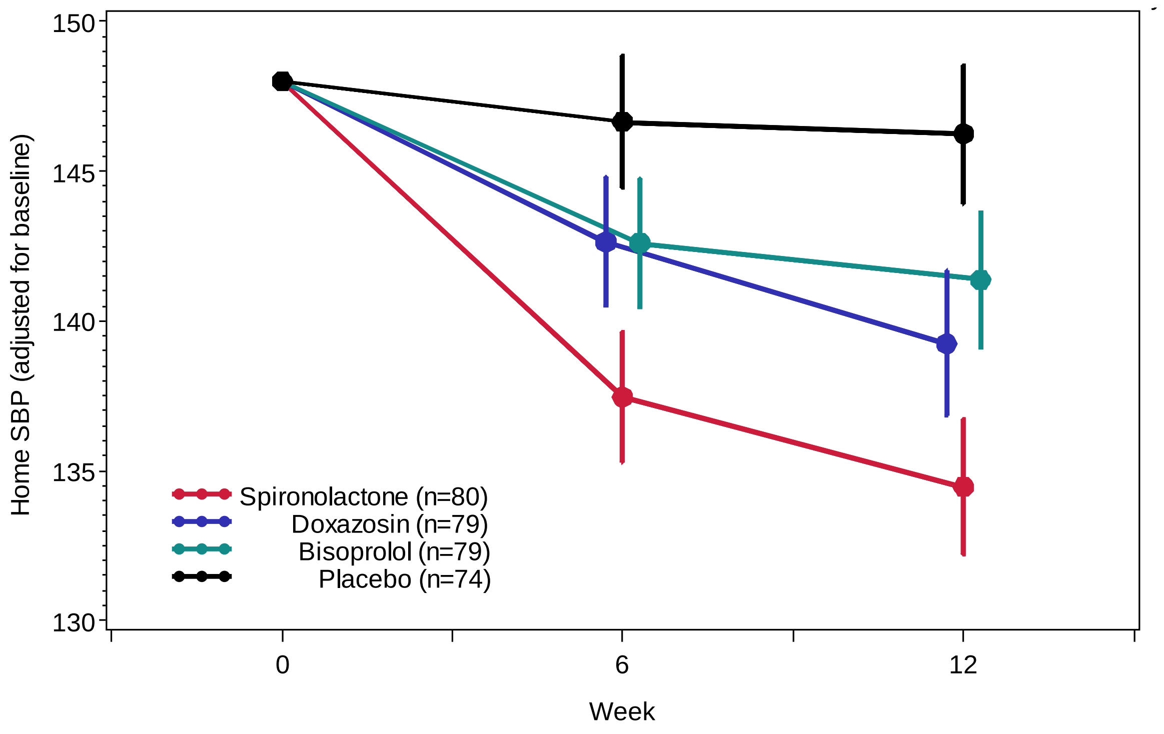

Least square means (95% CI) adjusted for baseline covariates in a mixed effects model

Supplementary Figure S4. **Prediction by plasma renin of best drug**

A. Prediction of best drug by any margin

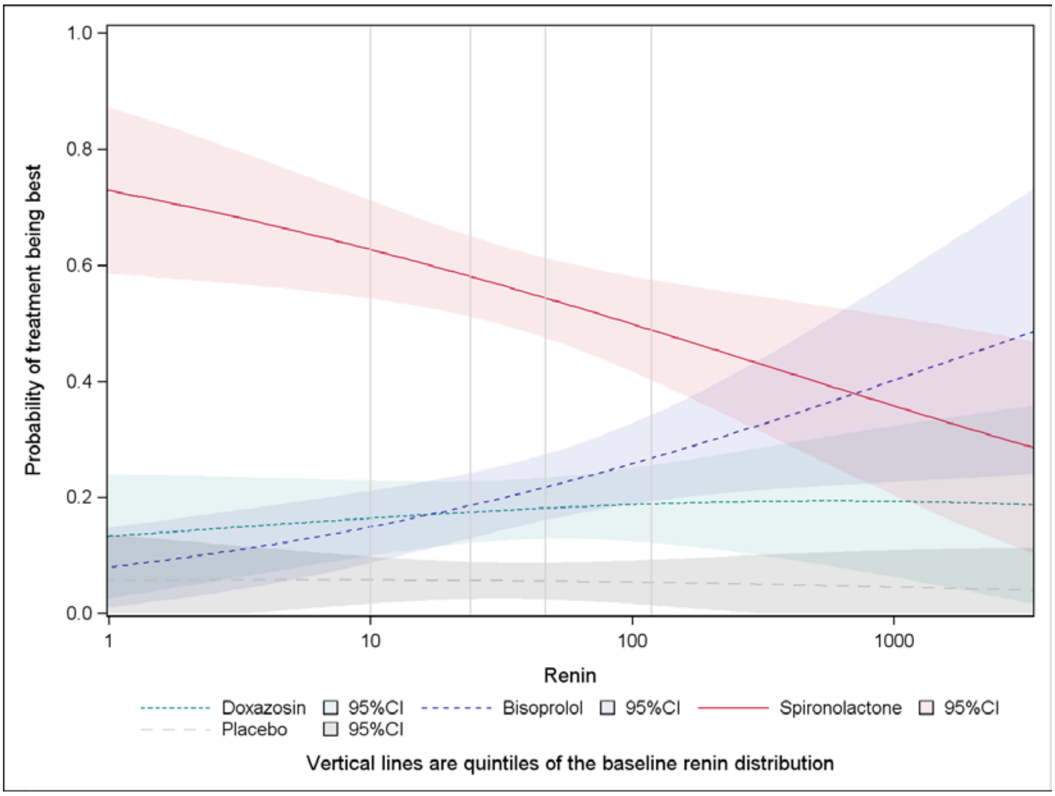

B. Prediction of best drug by >= 5mmHg

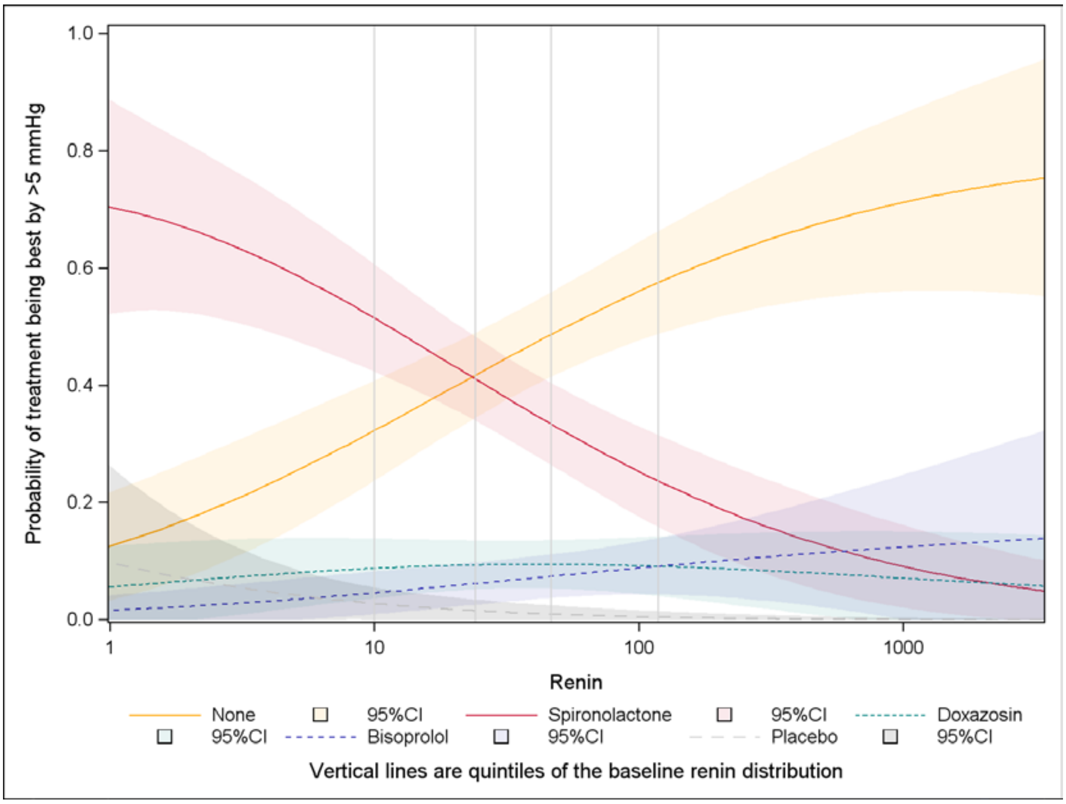

Probability of treatment being best by >5 mmHg
1.0
0.8
0.6
0.4
0.2
0.0
1
10
100
1000
Renin
None
95%CI
Spironolactone
95%CI
Doxazosin
95%CI
Bisoprolol
95%CI
Placebo
95%CI
Vertical lines are quintiles of the baseline renin distribution

Supplementary Figure S5. Frequency histogram for baseline plasma renin

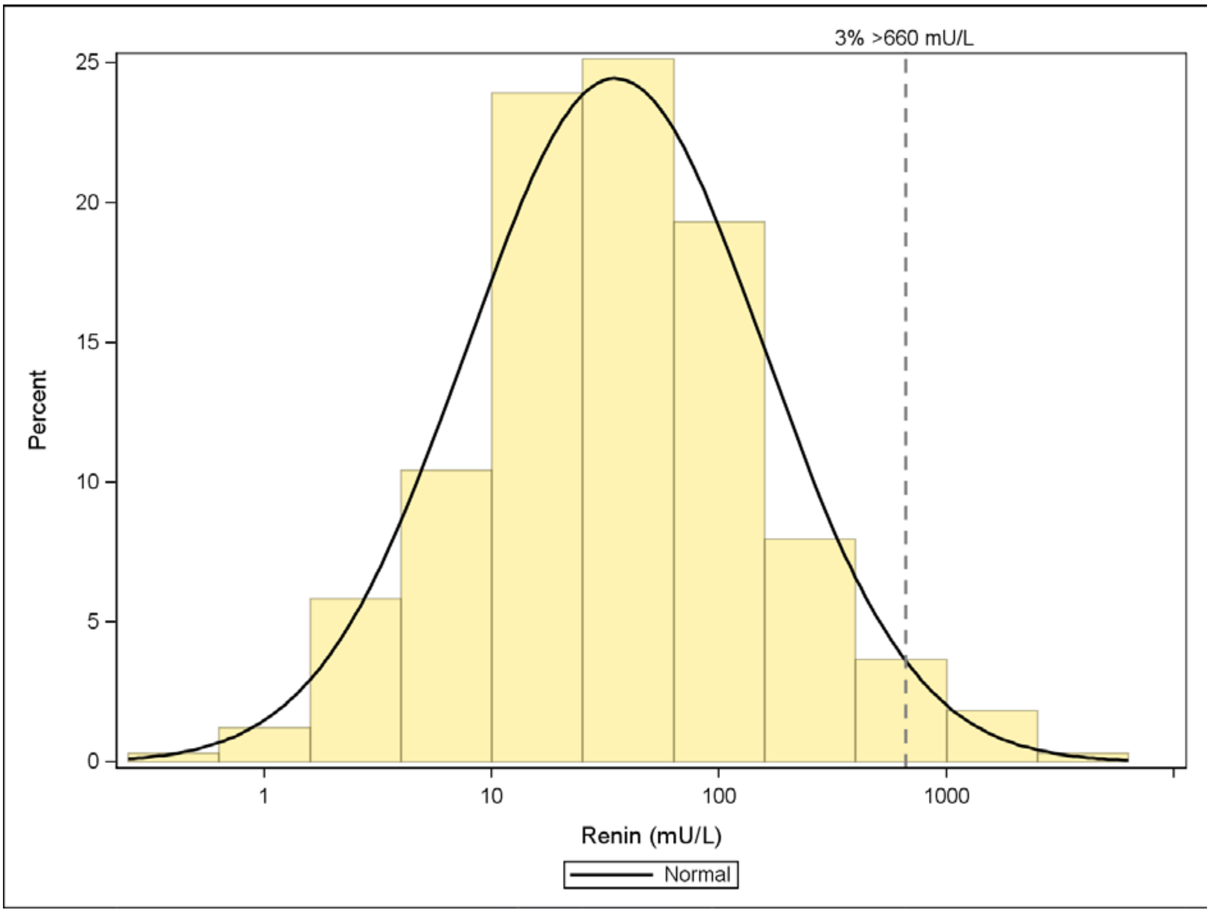

The dashed vertical line shows the renin value above which the fit lines for prediction by renin of HSBP change on spironolactone and bisoprolol intersect (see Figure 2b)
